# Desired versus actual delivery route: nursing students’ perception about their type of delivery

**DOI:** 10.1590/1980-220X-REEUSP-2022-0217en

**Published:** 2022-12-05

**Authors:** Camila da Silva Pereira, Jéssica Lima Soares, Thaís Isidório Cruz Bráulio, Simone Soares Damasceno, Dayanne Rakelly de Oliveira, Rachel de Sá Barreto Luna Callou Cruz

**Affiliations:** 1 Universidade Regional do Cariri, Departamento de Enfermagem, Crato, CE, Brazil.; 2 Universidade Regional do Cariri, Programa de Pós-graduação em Enfermagem, Crato, CE, Brazil.

**Keywords:** Parturition, Students, Nursing, Obstetric Nursing, Mothers, Humanization of Assistance, Qualitative Research, Parto, Estudiantes de Enfermería, Enfermería Obstétrica, Madres, Humanización de la Atención, Investigación Cualitativa, Parto, Estudantes de Enfermagem, Enfermagem Obstétrica, Mães, Humanização da Assistência, Pesquisa Qualitativa

## Abstract

**Objective:**

To identify the perception of nursing student mothers regarding the desired type of delivery in relation to the one performed.

**Method:**

Qualitative research, supported by the assumptions of Humanization of Obstetric Care, developed remotely with 16 nursing students who experienced the parturition process. The data were collected through an online focus group using the Google Meet program, guided by a semi-structured script. The speeches were analyzed using the Thematic Content Analysis technique.

**Results:**

An unsatisfactory discrepancy was identified between the mother’s preference and the realization, predominantly the desire for the vaginal way due to academic knowledge about the benefits. However, the cesarean section was predominant as a procedure performed due to possible complications, fears, financial situation of the parturient woman and control of choice by the health professional.

**Conclusion:**

Perception indicates marked disagreements, with the presence of complications, fear, lack of autonomy and predominance of a hegemonic care model, reinforcing the need for expansion and application of the Humanization of Obstetric Care, as a primary condition for proper monitoring.

## RESUMO

**Objetivo:**

Identificar a percepção de mães acadêmicas de enfermagem quanto ao tipo de parto almejado em relação ao realizado.

**Método:**

Pesquisa qualitativa, apoiada nos pressupostos da Humanização da Assistência Obstétrica, desenvolvida de forma remota com 16 acadêmicas de enfermagem, que vivenciaram o processo de parturição. Procedeu-se a coleta por meio de grupo focal *online* pelo programa *Google Meet,* guiado por roteiro semiestruturado. Os discursos foram analisados com base na técnica de Análise Temática de Conteúdo.

**Resultados:**

Identificou-se discrepância insatisfatória entre a preferência da mãe e a realização, predominando-se o desejo pela via vaginal em virtude dos conhecimentos acadêmicos acerca dos benefícios. No entanto, a cesariana se sobressaiu enquanto procedimento realizado em razão de eventuais complicações, medos, situação financeira da parturiente e controle da escolha pelo profissional de saúde.

**Conclusão:**

A percepção indica discordâncias marcantes, com a presença de complicações, medo, ausência de autonomia e predominância de um modelo assistencial hegemônico, reforçando a necessidade de ampliação e aplicação da Humanização da Assistência Obstétrica, como condição primária para um adequado acompanhamento.

## RESUMEN

**Objetivo:**

Identificar la percepción de madres estudiantes de enfermería en cuanto al tipo de parto deseado comparado con el realizado.

**Método:**

Investigación cualitativa, apoyada en los programas de Humanización de la Asistencia Obstétrica, desarrollada de forma remota con 16 estudiantes de enfermería, que vivieron el proceso de participación. La recolección de datos se realiza a través de un grupo focal en línea mediante el programa Google Meet, guiado por un guión semiestructurado. Los discursos fueron analizados con base en la técnica de Análisis Temático del Contenido.

**Resultados:**

Se identificó una discrepancia insatisfactoria entre la preferencia de la madre y la realización, predominando el deseo de la vía vaginal en virtud de los conocimientos académicos sobre los beneficios. Por lo tanto, la cesárea fue predominante como procedimiento realizado en razón de las eventuales complicaciones, los medicamentos, la situación financiera de la parturienta y el control de la elección por parte del profesional de la salud.

**Conclusión:**

La percepción indica discordancias marcadas, con la presencia de complicaciones, medo, ausencia de autonomía y predominio de un modelo asistencial hegemónico, reforzando la necesidad de ampliación y aplicación de la Humanización de la Asistencia Obstétrica, como condición primordial para un adecuado acompañamiento.

## INTRODUCTION

The experience of giving birth is tantamount with its importance in the life trajectory of women, and is characterized as a unique moment that may be remembered in their lifetime^(1)^. Predominantly in history, many go through the birth process, which is understood as a natural phenomenon that should happen in a favorable and noninvasive way, being the mother the protagonist of the process, participating actively and autonomously^(2)^.

With the institutionalization and growing use of technologies in the conduction of deliveries, cesarean sections (C-sections) have been encouraged as a safe, faster and painless option. This indication rapidly contributed to the increase in the rates of this type of delivery, changing not only the scenario, but the scene, with the hospital becoming the environment considered safe and the medical professional the main actor^(3)^.

However, the right to choose the route of delivery belongs to the woman. The focus on female empowerment presupposes the conscious and informed choice of the route of delivery. To this end, nurses are essential in the effectiveness of the female protagonism regarding the route of delivery, by promoting information that can help her choose and offer dignified care, thus contributing to positive experiences and maternal satisfaction^(4)^.

The perceptions, expectations and motivations of each pregnant woman should be valued, since they cover physical, emotional and sociocultural aspects that need to be respected in the individuality and wholeness of each being. These aspects can influence not only the choice of the type of delivery, but also the determination and way of facing the process^(5)^.

In this context, it is evident the need for elements of health promotion in obstetric care, improving the look aimed at the experiences of those who have undergone the birth process, especially those who will have a major participation in the delivery of other women, assisting in choices and care strategies that can meet the individual needs of each parturient^(6)^, as is the case of nursing students. However, studies that analyze perceptions about childbirth include puerperae in the immediate postpartum period as target audience without any criteria related to educational characteristics, nor articulate the perceptions to desires and expectations regarding the type of delivery^(7,8,9,10)^.

In the literature, there is a scarcity of studies addressing the experiences of nursing students and nurses about the type of delivery. The focus of publications has been on the experiences of women assisted by nurses during labor, highlighting the role of nurses in the process^(10)^, reinforcing the need to expand the debate to this specific audience, especially because they will be future nurses and, when they have the correct knowledge and skills, their actions in labor and birth care can be decisive for the consolidation of the principles of humanization of obstetric care.

It is believed that having women as the protagonists of their experiences favors autonomy and the valorization of their individual characteristics during the entire process of parturition. Taking this context into account, the present study aimed to identify the perception of nursing student-mothers regarding the type of birth desired in relation to the one actually performed.

## METHOD

### Type of Study

A descriptive study with a qualitative approach, based on the Consolidated Criteria for Reporting Qualitative Research (COREQ). The assumptions of the Humanization of Obstetric Care were used as a theoretical reference, considering that its precepts are related to improvements in the monitoring of women throughout the pregnancy-puerperal cycle^(11)^. These assumptions are based on the right to access, dignified and quality care during pregnancy, childbirth and puerperium, adequate prenatal care, the right to know and have assured access to the maternity hospital where they will be assisted at the time of delivery, childbirth and puerperium care performed in a humanized and safe way, as well as neonatal care^(12)^. In short, it is prioritized the care with dignity and adoption of beneficial actions in monitoring the birth process, avoiding interventionist and invasive practices that confer great risks to the mother-child binomial^(13)^.

### Population

The study population was composed of undergraduate nursing students who were regularly enrolled in the higher education institution where the research was carried out.

### Data Collection Site

The research was conducted virtually in all semesters of the nursing course of a public university located in the Cariri region, south of the state of Ceara, Brazil, conducted remotely due to the pandemic of COVID-19 and its rapid and high rate of transmissibility^(14)^.

### Selection Criteria

Twenty-one nursing students, at least 18 years old and who had already gone through the parturition process, were considered eligible to participate in the study. These students were selected from a survey of students who had already given birth, requested by means of a letter to the nursing academic center of the study institution, with prior authorization from the course coordination. We excluded five students who had limitations in access to the internet that hindered or made it impossible for them to understand and participate in the meetings; therefore, 16 students participated in this study.

### Data Collection

Data collection was done during the months of July and August 2020, by the main researcher, a nursing student, who participated in orientations about qualitative research, and by the project mentor, a doctoral student and researcher in the field of maternal and child health. For the participants of the study, a Google Forms link in questionnaire format was sent by e-mail to inform about the purpose of the study and collect information such as socio-demographic data, obstetric history and number of pregnancies, deliveries and occurrence of abortions.

We chose to collect information about the participants’ experiences regarding the type of delivery through two online Focus Group (FG) sessions, with an average duration of 1 hour and 30 minutes, and a total of eight students in each meeting, organized according to the participants’ availability. The FG is considered a tool capable of generating data with good functionality, contributing to actions aimed at well-being and quality of life, being commonly used in qualitative research^(15)^.

To effectively conduct the FG sessions, invitations were previously sent through links to access e-mails, as well as reminders before each group. The FG sessions were conducted via the Internet through the Google Meet program version 2020, using a semi-structured script, containing the following key questions directed to the focus of the study: what type of delivery was performed and how did this experience occur? Were there differences between the type of delivery that was performed and the type of delivery desired during pregnancy? Did being a nursing student interfere in the choice of the type of delivery? The criterion for ending the collection was based on data saturation, i.e., when the listed objective was effectively answered, being verified the redundancy in the findings^(16)^.

It is noteworthy that a pre-test of the script that guided the FG sessions was performed with obstetric nursing residents of the same institution, who had already experienced the birth process, allowing to verify gaps and make changes and adjustments necessary for the application of such script.

When the FG sessions, scheduled with the participants began, the researchers formally introduced themselves to the students, who were first oriented about the objective to be reached with the study, as well as the motivations of the researchers to proceed with the research and the benefits to be achieved. To better conduct the groups, it was pointed out the importance of the students discussing their last experience, for the case of those who might have more than one child, therefore, warned about the confidentiality of the information provided.

At the time of the speeches, the mediators/facilitators – a nursing student and a doctor in nursing who had previously studied the application of focus groups and participated in the pre-test and readjustment of the script used - allowed the participants to talk comfortably about their type of birth, directing them to keep the focus on the triggering questions contained in the script.

In the FG sessions, a master’s student in nursing was also present to observe and report, recording the speeches, facial expressions, and the most relevant aspects of the discussion, and controlling the online audiovisual tools. The sessions followed the methodological assumptions of Minayo and Costa^(16)^.

### Data Analysis and Processing

The speeches from the focus group sessions were recorded by the very communication program Google Meet version 2020, then the material was rigorously transcribed, highlighting the main ideas expressed, then the material returned to the participants after transcription, in order to obtain validation.

Subsequently, such data were managed manually and organized based on the Thematic Content Analysis technique, which refers to a method of qualitative data analysis to identify, analyze, interpret and report patterns bringing detailed explanations of the method following three steps: pre-analysis, exploration of the material and treatment of the results obtained and interpretation^(17)^. The sociodemographic data were analyzed by descriptive statistics. After the analysis process, the students’ reports were discussed using the lens of the premises of the Humanization of Obstetric Care.

From the transcribed speeches of the participants, an individual coding was applied, using the acronym for nursing student (AE) and the order of entry into the group session, for example, AE1, AE2, AE3 and so on, as a representative form of their participation, thus safeguarding their anonymity.

### Ethical Aspects

The research in question was appreciated by the Ethics and Research Committee of the Regional University of Cariri, being approved in 2020 by opinion no. 4,050,608, meeting the Resolution of the National Health Council no. 466/12 that covers the ethical aspects of research involving human beings. After approaching the participants in order to agree to participate in the research, and the clarification of its objectives, electronic acceptance was requested through an online form of the Informed Consent Form (ICF), starting the collection only after this consent.

## RESULTS

The profile of the 16 participants revealed a mean age of 28.3 years, with most living in the city where the institution where they were enrolled is located, being 31.25% (n = 5) in the eighth semester, 18.75% (n = 3) in the tenth, 12.5% (n = 2) in the fourth, sixth and ninth and 6.25% (n = 1) in the first and fifth semester. Other sociodemographic and obstetric characteristics are presented in Table 1.

**Table 1.** Sociodemographic and obstetric characterization of nursing students (n = 16) – Crato, CE, Brazil, 2020.

Variable	N	%
Race		
Brown	8	50%
White	7	43,7%
Yellow	1	6,3%
Marital status		
Single	7	43,7%
Common law	5	31,25%
Divorced	3	18,75%
Married	1	6,25%
Religion		
Catholic	11	68,75%
Evangelical	3	18,75%
Atheist	2	12,5%
Work		
Has a profession	9	56,2%
Works in that profession	7	44,4%
Number of pregnancies		
One	8	50%
Two or more	8	50%
Type of birth		
Vaginal	5	31,25%
C-section	11	68,75%
Abortion		
Yes	3	18,75%
No	13	81,25%

**Chart 1.**
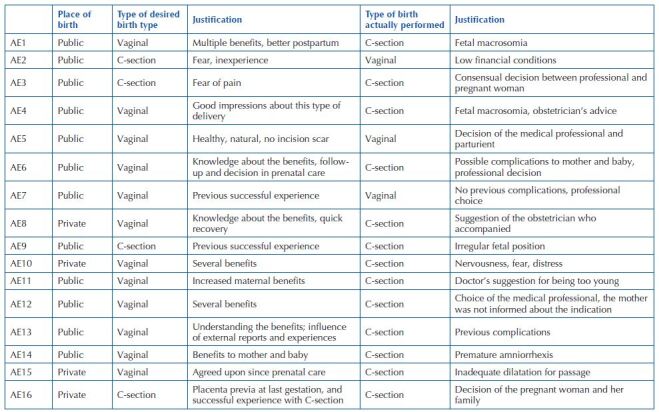
Place of childbirth care, type of desired and actual delivery of the participants (n = 16), with respective justifications – Crato, CE, Brazil, 2020.

The summary of the place of childbirth care, type of desired delivery and the type of delivery actually performed, with respective justifications, are shown in Chart 1.

The students’ perceptions and expectations during pregnancy and childbirth were organized through the reports into thematic categories, namely: “Type of delivery performed”, “Differences between the type of delivery that was performed and the type of delivery desired during pregnancy”, and “Did being a nursing student interfere in the choice of type of delivery?”.

### Type of Delivery Performed

The nursing student mothers were able to express perceptions about their type of delivery in the FG sessions, where they were initially asked about the type of delivery they had, inducing them to narrate about this experience. The following are the students’ reports. *My delivery was normal, I spent a period of more than nine hours with ruptured membranes, I arrived with high blood pressure, but anyway, by the time I had my son, it was painful, as all normal births are painful. But it is very good, I didn’t have much experience, so, we also, at that time, didn’t have much information. But I think it was natural, it was good, I have no scar from the surgical incision and my delivery was healthy, despite the delay* (AE5).

It can be seen in the report that the mother (AE5) mentioned delay, some complications and perceived labor as a painful process, however, even in the face of such issues, she understood that she had a positive and healthy experience. Other mothers (AE6 and AE14) then showed negative experiences in childbirth, pointing out suffering and delay.

*At the time of delivery, I was feeling slight contractions, and then when I got there, they attended to me and sent me home, when I left the maternity unit my bag broke, they took me inside the hospital and there I stayed waiting. In short, I went to the operating room alone, at 4:30am my son was born, on Sunday. He was born very purple, he had swallowed a little liquid, but was born well, I remember that she said that his APGAR was good, so my experience regarding delivery I didn’t like it very much because of the delay, I think they let me suffer too much, until I decided which delivery I was going to have (AE6).*

*When the other doctor’s shift changed, they came to perform a cesarean delivery, they had difficulty in auscultating the baby’s heart, so I was scared, desperate, but it worked! It was a little traumatic for me (AE14).*

While expressing their perceptions, some students revealed that they had successful experiences with the delivery they had given birth to.

*I was super calm, I didn’t feel anything on the day, simply nothing, my bag didn’t break, my dream was that my bag would break, in the middle of the street, wherever! But it didn’t, and I was disappointed. I went through the C-section surgery, it was calm, I had all the assistance from the professionals (AE8). The surgery was very calm, fast, only the anesthesia, that the first one the doctor didn’t get, but in the second one she got it, thanks God, and I think that in less than five minutes my baby was crying (AE4). It was easy in relation to childbirth (…) it was also through SUS* ^*1*^ *and the experience was good (AE13).*

### Differences between the Type of Delivery that was Performed and the Type of delivery Desired during Pregnancy

When asked if there were differences between the type of delivery that was performed and the type of delivery that was desired during pregnancy, some participants mentioned discrepancies, both due to financial issues and to complications and the influence of other people regarding their choice.

*My delivery, from the beginning I wanted it to be a normal, natural birth, but due to all the complications, it was not possible (AE8). I didn’t have much of a choice, I couldn’t pay privately and was left in the hands of SUS (AE2). Well, in my case, when we start prenatal care here we don’t have much choice, they only send you for a c-section when they see that the baby or you have some complication, in my case it wasn’t like that, so I can’t say that I had a choice, it was a normal birth (AE7).*

*I wanted a normal delivery but when I arrived at the hospital, I had no information about what type of delivery would be, so much so that I arrived in labor, but after a while the doctor came to tell me that I had to have a cesarean section, but the reason was not communicated to me (AE12). My mother, my husband and the doctor made the decision. I had the dream of having a normal delivery, I thought it would be better for me (…) they said I was too young and it was too much suffering (AE11).*

Added to the fact that the delivery was not the desired one, one participant (AE1) reported that, in view of the pandemic, her experience was not what she had hoped for.

*Yes, I intended to have the baby in a normal delivery, but I dilated and there was no way down, so an emergency C-section had to be done because the baby was already starting to suffer, and for me it was a bit of a bad experience, because I started to feel alone too, because of this pandemic, so it was just me there in the room feeling the pain, and it’s very bad to be alone, horrible! (AE1)*

### Did Being a Nursing Student Interfere in the Choice of Type of Delivery?

The students were also asked if being a nursing student interfered in their choice of type of delivery.

*Yes, yes, it did! Because you are from the health area and have more knowledge than other people who are not, then you know that a normal birth is good for you because of the postpartum period, it’s good for the baby. So, from the knowledge that I had, I saw that normal birth would really be the best choice, but unfortunately the circumstances didn’t allow it (AE1). From the choice itself and the whole path I took during pregnancy until the moment of delivery, yes! (AE15).*

*It was relevant in the question of knowledge, the more knowledge, the more security about what you really want (AE16). Yes! I was even pregnant with her during the women’s health course, so I think it influenced a lot! (AE3). As I had already observed the experience of other women in labor and the suffering they also had during the internships, this influenced my decision a lot, for sure! (AE10).*

As student-mothers, the practice at the university revealed a fearful look at the birthing process, deepening a divergent perception of the choice of the route studied as ideal.

*I think that for us nursing students, when we witness the childbirth process and the process of pain a lot, I think that it distances us more and more from the process of normal birth, I think it’s even ugly to say this, but it’s true, because we see at university that normal birth is the most suitable, it’s the best birth, it’s the best for the mother, it’s the best for the baby, it’s everything! But, as we experience very closely the process of pain that the patients go through, the suffering until the baby is born, I think this take us apart a lot. If I were to have a third child, which is not in my plans, then I wouldn’t really want a normal birth (AE3). I was seven months and I was dealing all the time with women giving birth, women having abortions, having C-sections of dead children, this really messed up my psychology (…) then I said: no! I’m going to C-section again (…) in normal birth. So many things can go wrong! (AE9).*

## DISCUSSION

The interpretative analysis of the participants’ speeches synthesized marked differences between the type of birth desired during pregnancy versus the one performed, revealing that the fact of being a nursing student and probably having more information on the subject was not able to overcome the barriers related to the lack of female protagonism and autonomy in choosing the route of delivery, fear and sovereignty of the medical indication for C-section, even when it was not a true obstetric indication, similar to what happens to other women in the general population.

Agreeing with what was exposed in this study, regarding the prominence of C-sections as a procedure performed, a Brazilian cross-sectional study reports that in the face of admissions of pregnant women for childbirth care, the mean rates of C-sections in the units surveyed ranged from 24.8% to 75.1%, drastically exceeding the 15% recommended by the World Health Organization (WHO), potentially leading to consequences of potential risk to mother and child^(18,19)^.

This study demonstrated that choosing the vaginal delivery route tends to be more desirable when compared to C-sections, because of the lower frequency of postpartum complications and various benefits to the binomial, possibly not interfering early in the mother-baby interaction. It is considered that factors associated with cesarean sections limit and also interfere in the first relationships of the newborn with the mother^(20)^.

The impossibility of choosing the desired route of delivery reported by some students is emphasized, justifying this lack of protagonism/autonomy by being in a public hospital unit and having low financial conditions. The choice of birth route is influenced by several factors, including cultural, social, individual, and economic issues. Women of lower income perceive themselves as having less power of choice, being subjected to unnecessary interventions. Conversely, women with high income perceive themselves more welcomed in their decision for the operative delivery route, but little informed during their follow-up^(21)^.

It is historically observed that the expansion of access to hospital delivery care in the Unified Health System (SUS) cooperated to the decrease in the occurrence of negative outcomes. However, challenges to the evolution of quality of care persist, as well as barriers in the connection between services at different levels of care^(22)^.

In this study, there was a predominance of choice of delivery route by the medical professional, and the mother in one of the cases was not informed about the indication. This reality is opposed to the principles of Humanization of Obstetric Care and constitutes a serious violation of the rights of women and children, which advocate that the interaction and care between professional and patient need to be established, and professionals are responsible for transmitting safety and respect to pregnant women and their families, maintaining a relationship of dialogue between the parties^(23)^.

Information, decision-making, and responsibilities must be shared between the professional and the parturient woman. Thus, decisions and choices need to be dialogued, and the pregnant woman has the right to make a conscious and informed choice, and the health professional, aware of the situation, should provide all necessary and requested information by the woman and her family, as a form of a shared and conscious decision-making process, thus promoting humanized care^(23,24)^.

It was occasionally identified the fear of pain, nervousness, loneliness, distress and delay in delivery interfering significantly in the choice of the type of delivery and consequently in the perception of each mother about the event experienced. Results from a Brazilian study corroborate this finding, indicating that childbirth is an event that generates various feelings in pregnant women, from extreme happiness to feelings of fear, anxiety and pain, thus contributing to increased rates of C-section operations^(25)^.

Faced with these events that show negative perceptions, the premises of humanized obstetric care allow opening to other modalities of care, such as the humanistic model, which presents technological and scientific advances of the technocratic model and the therapeutic possibilities of the holistic model, allowing the use of low-cost non-pharmacological methods for pain relief such as massage, use of the Swiss ball and warm baths, respecting, above all, the physiology of each woman^(11,23)^.

Even with the appropriate use of technologies that can be made available to pregnant women in labor, it is necessary to think of the parturient woman as a relational subject, which is linked to the social and family context, thus, as a way to promote comfort supported by legislation, the presence of a companion chosen by the pregnant woman during and after delivery is encouraged^(23–26)^. However, despite the importance of the presence of a companion providing emotional support, reducing the amount of C-sections, improving the physiology of childbirth and the postpartum period of the puerperal woman, the absence of the right to choose the companion is still noted in some hospital institutions that attend childbirth^(27)^.

As nursing students, the undergraduate students are taught about which route of delivery provides greater benefits to the mother and baby, and the key role of the humanization of childbirth in assistance, as a way to improve the care and assistance to women. However, when facing pregnancy, childbirth and puerperium care, still as a student in internship practices, unpleasant experiences of the obstetric context can be incorporated into the memory of students as they witness unnecessary interventions, suffering in childbirth, failures of the health system to maintain professional standards, insufficient support policies and other situations that contradict what was learned in the undergraduate course, influencing the student’s future childbirth choices^(28)^.

It is observed that, in practice, there is no overcoming of a hegemonic model of obstetric care, which favors the medicalization of problems and individualism in care^(29)^. Although these student mothers and future health professionals have qualified knowledge, they could not act in a way to have a leading role in their childbirth experiences.

Among the limitations of this study, we highlight the small number of publications available on the perceptions or experiences related to the childbirth process whose target audience were nursing students. Added to this, some limiting circumstances related to the collection, in which the participation of the interviewees by remote means occasionally led to failures in keeping the webcam on, but which were minimized by maintaining effective participation in the FG sessions through voice with the meet platform microphone on. Despite the above, it is understood that this means of collection and interaction ensured greater protection for both researchers and participants, given the pandemic situation experienced in the period in question.

However, it is considered that the results obtained through this research contribute directly to the advancement of scientific knowledge, since they are in agreement with the WHO directives, which encourages research that addresses the psychological and social aspects related to the type of delivery.

It is understood that the study provides implications for nursing, in the sense of bringing reflections about the student mother, as a future health professional who may work in the assistance to pregnant women, reflecting and guiding these women about the possibilities of a safe, healthy, respectful birth that encourages the autonomy of pregnant women in a process of extreme protagonism of the mother and the child.

## CONCLUSION

The perception of most students indicates dissatisfaction related to unmet expectations about the type of delivery desired during pregnancy. There was a predominance of desire for the vaginal route due to the influence of academic knowledge acquired about the benefits for the mother-baby binomial. While, in opposition to the expressed desire, the operative delivery route stood out as a procedure performed due to the singularity of possible complications pointed out by the professional, fears, unfavorable financial situation, lack of maternal protagonism and control of the choice by the doctor.

These findings show that the model of childbirth care still has hegemonic predominance, and in this model the woman does not have the protagonism that is recommended by good practices, even though these women are holders of knowledge, as in the case of students in this study. In this sense, there is a need for expansion of educational processes, emphasizing the Humanization of Obstetric Care as a primary condition for proper monitoring, aiming at improvements in the delivery process and, consequently, satisfactory experiences.

## Footnotes

^1^SUS: Brazilian Unified Health System
